# Effect of Cold Smoking and Natural Antioxidants on Quality Traits, Safety and Shelf Life of Farmed Meagre (*Argyrosomus regius*) Fillets, as a Strategy to Diversify Aquaculture Products

**DOI:** 10.3390/foods10112522

**Published:** 2021-10-21

**Authors:** Concetta Maria Messina, Rosaria Arena, Giovanna Ficano, Mariano Randazzo, Maria Morghese, Laura La Barbera, Saloua Sadok, Andrea Santulli

**Affiliations:** 1 Dipartimento di Scienze della Terra e del Mare DiSTeM, Laboratorio di Biochimica Marina ed Ecotossicologia, Università degli Studi di Palermo, Via G. Barlotta 4, 91100 Trapani, Italy; rosaria.arena@unipa.it (R.A.); giovanna.ficano@unipa.it (G.F.); maria.morghese@unipa.it (M.M.); andrea.santulli@unipa.it (A.S.); 2 Istituto di Biologia Marina, Consorzio Universitario della Provincia di Trapani, Via G. Barlotta 4, 91100 Trapani, Italy; mariano.randazzo@tin.it (M.R.); labarbera@consunitp.it (L.L.B.); 3 Laboratory of Blue Biotechnology & Aquatic Bioproducts (B3Aqua), Institut National des Sciences et Technologies de la Mer (INSTM), Annexe La Goulette Port de Pêche, La Goulette 2060, Tunisia; salwa.sadok@instm.rnrt.tn

**Keywords:** meagre, *Argyrosomus regius*, cold smoking, natural antioxidants, halophyte, aquaculture, value-added food product, shelf-life, fillets, fish quality

## Abstract

Aquaculture has been playing a leading role over the years to satisfy the global growing demand for seafood. Moreover, innovative techniques are necessary to increase the competitiveness, sustainability and profitability of the seafood production chain, exploiting new species from the aquaculture, such as meagre (*Argyrosomus regius*), to develop value-added products and diversify their production. In the present work, the effectiveness of cold smoking combined with antioxidants (SA) compared to cold smoking alone (S) on meagre fillets, the quality and shelf life were investigated. Sensory, biochemical, physical–chemical and microbiological analyses were performed on the smoked fillets during vacuum-packaged storage for 35 days at 4 ± 0.5 °C. The results showed positive effects of the SA treatment on the biochemical parameters of meagre fillets. The total volatile basic nitrogen (TVB-N) in smoked meagre fillets was significantly lower in the SA treatment at the end of storage compared to the S treatment. Moreover, SA had a positive effect on lipid peroxidation. Lower values of malondialdehyde (mg MDA/kg) were observed in the SA treatment during preservation compared to the S treatment. This work will contribute to the growth of the fish production chain, producing a value-added fish product by exploiting meagre, whose production has been increasing over decades.

## 1. Introduction

Global fish consumption has been increasing over the years due to the increasing world population, as well as the awareness of the beneficial effects of fish inclusion in a balanced diet on human health. It has been reported that the average annual rate at which global fish consumption increased from 1961 to 2017 accounted for 3.1%, almost twice that of the annual growth of the global population [1].

In this context, the fish production sector has grown over the years in order to satisfy the global growing demand for seafood, and it is foreseen to increase in the future. Specifically, the production of capture fisheries has not been increasing since the late 1980s, while aquaculture has been playing a leading role in satisfying the seafood demand over the years. In this scenario, the aquaculture contribution to fish production has constantly increased at the global level, reaching 46.0% in the period 2016–2018. In 2018, aquaculture accounted for 52% of the total production of seafood products that were used for human consumption. Specifically, in 2018, aquaculture produced 82.5 million tons of fish and other organisms, 32.4 million tons of plant organisms and a good amount of products not intended for consumption (26,000 tons) [1].

Innovation plays the major role in increasing competitiveness, sustainability and profitability of the seafood production chain, paying attention to develop value-added processed products by exploiting new species coming from aquaculture, an ever-increasing food production sector. This strategy would allow to satisfy consumers’ preferences in the food sector, who prefer ready-to-eat products with high nutritional value whose availability does not determine a negative effect on the environment and whose origin and quality are guaranteed [2]. In this context, meagre (*Argyrosomus regius*) represents an aquaculture species that must be considered and valorized, considering that its production in Europe has been increasing over the years. Specifically, meagre production has been rising progressively in Europe over the decades; in 2018, the production of meagre reached 6.827 tons (+270%), out of which 59% was covered by Spain [3].

Meagre, which belongs to the *Sciaenidae*, is a euryhaline fish spread in the Mediterranean Sea. Thanks to some characteristics of *A. regius*, such as its high adaptability to several environmental conditions and high resilience to stressors, it has been considered suitable for the diversification of the Mediterranean aquaculture [4]. In addition to these aspects, *A. regius* grows rapidly and reaches commercial size (700–1200 g) only after 12 months and 2–2.5 kg after 24 months under optimal conditions [4]. Moreover, meagre is characterized by a high nutritional value thanks to its flesh, lean and with a high lipid quality [5].

Fish is the main dietary source of omega-3 polyunsaturated fatty acids (PUFAs), highly beneficial for human health but also extremely prone to peroxidation, such as docosahexaenoic acid (DHA) and eicosapentaenoic acid (EPA). In particular, their oxidation generates free radicals that trigger chain reactions that eventually lead to the generation of compounds responsible for extremely unpleasant odors, as well as the loss of compounds of very high nutritional value [6,7]. Considering the characteristics of the raw material processed, highly perishable, preservatives should be used to preserve the fish quality and extend the shelf life; indeed, even if fish is preserved by freezing or cold storage to extend its shelf life, this would not be sufficient to prevent fish spoilage, lipid oxidation and rancidity with negative effects in terms of consumer acceptability.

Chemical preservatives have been used in order to prevent fish spoilage and extend the fish shelf life, such as sodium acetate, sodium lactate and sodium citrate, whose effects were investigated on extending the shelf life of salmon (*Onchorhynchus nerka*) during refrigerated storage [8], highlighting that using these preservatives was effective on extending seafood shelf life. Sodium bisulfite is also used as a chemical preservative to reduce enzyme activity and microbial degradation in shrimp (*Litopenaeus vannamei*) for 4–6 days; nevertheless, it has been reported that consuming food treated with sodium bisulfite for a long time can lead to a serious health crisis [9].

Therefore, the food industry has been prompt about consumers’ concerns and preferences for natural preservatives for the use of natural antioxidants and antimicrobials instead of synthetic ones [7]. Marine and terrestrial species have thus been used for the purpose of extracting antioxidant compounds, such as ascorbic acid, glutathione, carotenoids, terpenes and phenolic compounds [7,10,11,12,13,14].

Among plant-derived compounds, there are essential oils, a complex mixture of volatile compounds produced as secondary metabolites in plants and that give them their characteristic odor. Essential oils have been used as natural preservatives for preserving fish quality and extending their shelf life due to their well-recognized antimicrobial and antioxidants potentials [15] Plant extracts could also been used for application in fish preservation for their antimicrobial and antifungal activities, as well as their capacity to inhibit lipid oxidation [7,16].

Salicornia strobilacea (*Halocnemum strobilaceum*) is a plant that belongs to halophytes and grows along salt marshes, salt lakes and saltworks all over the world. Due to the extreme environmental conditions under which this kind of plant grows, it is characterized by powerful antioxidant systems that employ several components, among which are the secretion and accumulation of polyphenols. These bioactive compounds were obtained from the plant (*H. strobilaceum*) and tested in vitro, showing strong antioxidant and antibacterial power [17].

The positive effects of antioxidants from *H. strobilaceum* when combined with other preservative techniques such as modified atmosphere packaging and cold smoking were shown previously on the sensory, physical–chemical, nutritional, biochemical and microbiological properties of dolphinfish (*Coryphaena hippurus*) fillets [18,19].

Cold smoking is a preservation technique also used to create new value-added products. Specifically, in cold smoking, after an initial treatment with salt, the product is smoked in order to give the product organoleptic characteristics appreciated by consumers, as well as to transfer to it antimicrobial and antioxidant compounds (aldehydes, ketones, alcohols and phenols), with a significantly longer shelf-life than fresh product. During the different phases of the process, the temperature is never higher than 30 or 33 °C [20,21]. To date, several fish species have been processed by this technique, such as farmed European sea bass (*Dicentrarchus labrax*) and Atlantic salmon (*Salmo salar*) [22,23]. Moreover, fishery species have been valorized by applying cold smoking in order to make available all year round seasonal species, as in the case of dolphinfish (*Coryphaena hippurus*), but also to enhance fishery species with excess catches, such as sardines (*Sardina pilchardus*) [19,24,25] and herring (*Clupea harengus*) [26].

The effectiveness of combining traditional techniques such as cold-smoking with the addition of natural preservatives on maintaining the quality and prolonging the shelf life of new value-added seafood products has been investigated [19,27]. Combining an antioxidant treatment with oregano extract and cold-smoking produced a reduction in sardine (*S. pilchardus*) lipid oxidation compared to traditional smoking, highlighting the effectiveness of the combined treatments on improving the shelf life of smoked fish [27].

The combination of cold smoking and natural preservatives was also investigated on dolphinfish (*C. hippurus*) fillets [19]. Overall, the combination of antioxidants with cold smoking showed positive effects on the quality of dolphinfish fillets, improving the biochemical, microbiological and sensory aspects of the product and, consequently, enhancing the marketability of *C. hippurus* and contributing to costal fishery sustainability.

The present work was aimed at studying how the combination of cold smoking and natural antioxidants affects the sensory, biochemical, physical–chemical and microbiological properties of meagre (*Argyrosomus regius*) fillets. As a source of antioxidants, *H. strobilaceum* extracts were used.

The present study will contribute to increasing the competitiveness, profitability and sustainability of the seafood production sector by developing a new value-added product by using an aquaculture species (*A. regius*) of high nutritional value whose production has been increasing over the years, contributing to diversifying seafood production, meeting consumers preferences and reducing the pressure on overexploited fishery resources.

## 2. Materials and Methods

### 2.1. Fish Sampling and Processing

A total of 20 specimens of *A. regius* (average size 27.17 ± 1.91 cm and average weight 347.72 ± 62.85 g) were processed in a Sicilian (Italy) aquaculture plant to obtain forty fillets (mean weight: 73.48 ± 22.49 g) that were stored on ice. In the laboratory (less than 30 min), the fillets were stored under vacuum in Foodsaver bags (HDPE) and nylon bags (http://www.gopack.it, accessed on 1 September 2021) and subjected to rapid freezing at −35 °C (AB 2/3 ALLFORFOODD (PU), Italy) and maintained in this condition for 24 h to inhibit the bacterial growth [28].

The next day, all samples were removed from the bags, placed in air-permeable LDPE bags and thawed at 4 °C for 8 h before processing.

### 2.2. Salting and Smoking

**Figure 1:**
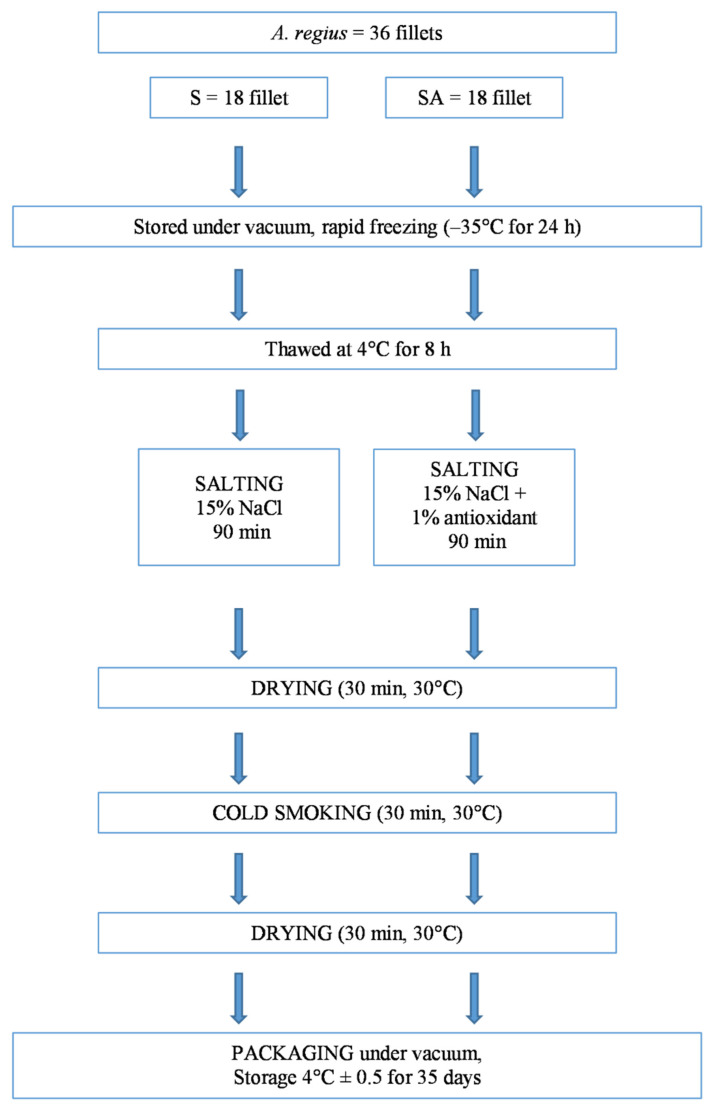
Experimental design flowchart. Cold smoking (S); Cold smoking combined with antioxidants (SA).

Thawed *A. regius* fillets were processed as follows: four thawed fillets were used for the analyses of the untreated product, and the remaining 36 fillets underwent a smoking process consisting of four steps: salting, first drying, smoking and second drying [19,22], as shown in Figure 1.

Thawed fillets were separated in 2 batches: in the first batch, 18 fillets were immersed in standard brine (S) consisting of a 15% NaCl solution (*w/v*); in the second batch, 18 fillets were treated with the same standard brine with 1% antioxidants (SA). For both treatments, a fillet:brine ratio of 1:4 was used [19,22].

The antioxidant solution was prepared as described by Messina et al. [18]. Briefly, dried and pulverized *Halocnemum strobilaceum* was extracted with distilled water (1:10 *w/v*) for 24 h. The sample was then filtered and lyophilized [29]. The final solution of *H. strobilaceum* was prepared by dissolving 10 g of freeze-dried extract in 1000 mL of distilled water, with a polyphenol content equal to 500-mg gallic acid equivalent (GAE)/L [18,19].

Brine salting was performed for 90 min; then, the samples were dried for 30 min at a temperature of 30 °C. The fillets were cold-smoked using Moduline oven model FA082E (Scubla srl, Remanzacco (Ud), Italy) for 30 min at 30 °C, as described by Messina et al. [19,22].

After cold smoking, the fillets were dried as described previously.

At the end of the process, all the fillets were sealed in vacuum bags and stored at 4 ± 0.5 °C for 35 days.

Three fillets from each treatment were analyzed at regular intervals (1, 7, 14, 21, 28 and 35 days after smoking). The effects of the smoking process combined with natural antioxidants on the quality of *A. regius* fillets were evaluated through a multidisciplinary approach involving sensory, physicochemical, biochemical and microbiological parameters.

Following sampling for the microbiological analysis and the sensory and instrumental analyses, the fillets were cold-homogenized for the analysis of the biochemical and nutritional parameters related to shelf life.

### 2.3. Physical–Chemical Parameters

#### 2.3.1. Color

Color readings were taken in the L*, a* and b* color space (CIELAB color space, D65 standard illumination and a 2° observer) and repeated 3 times using a Konica Minolta colorimeter (Osaka, Japan). The evaluated color parameters were lightness (L*), red–green chromaticity (a*), yellow–blue chromaticity (b*), the saturation or intensity of the color chroma (C*) and the hue angle (h), as recommended by the International Commission on Illumination [30,31,32]. The analyses were performed in triplicate for each sample. The color evaluation was carried out in two dorsal regions of the fillets along the cephalocaudal direction.

#### 2.3.2. Texture Profile Analysis

The texture analysis was conducted as described by Messina et al. [33]. Two small fragments (1.8 cm Ø) obtained from the same portions of each fillet were used. The analysis was performed at room temperature using Instron Texture Analyzer Mod. 3342 (Turin, Italy). The measured parameters were hardness (N) and the Young’s modulus or modulus of deformability (N/mm^2^) (i.e., the force and the slope of the curve at 50% compression, respectively) [19,34]. The analysis was performed in triplicate. In particular, for each replicate mentioned above, two fragments per fillet along the dorsal margin were considered. The samples were maintained in ice before the analysis.

#### 2.3.3. Water Holding Capacity (WHC)

The Water holding capacity (WHC) was determined using the method described by Teixeira et al. [35], with some modifications reported by Messina et al. [33]. The analyses were performed in triplicate for each sample. The results were expressed in percentage (% WHC).

#### 2.3.4. Muscular pH

The muscular pH of the fillet was measured at three points along the lateral line with a Crison pH meter (Barcelona, Spain) equipped with a BlueLine, pH 21 Schott Instruments (Weilheim, Germany) combined electrode.

#### 2.3.5. Water Activity Determination

The water activity (aw) was measured with a fast water activity meter (HP23-AW Rotronic, AG, Bassersdorf, Switzerland). The temperature at which the water activity was measured was equal to 21 ℃. The analysis was performed in triplicate.

### 2.4. Proximate Composition and Biochemical Parameters Related to the Shelf-Life

#### 2.4.1. Proximate Composition

After the physical–chemical and sensory analyses, in order to carry out further analyses, the fillets were cold-homogenized.

The ash (ignition at 600 °C for 5 h) and moisture (drying at 105 °C for 24 h) contents (% ash and moisture) were assessed according to the AOAC method [36]. The protein content (% protein) was determined according to the AOAC method [37].

The total lipids (% lipids) were determined according to Folch et al. [38], and the fatty acid (FA) methyl esters (%) were determined by the method of Lepage and Roy [39]; gas-chromatography was carried out following the operating conditions described by Messina et al. [40].

#### 2.4.2. Biochemical Parameters Related to the Shelf-Life

The production of thiobarbituric acid reactive substances (TBARS) was determined using the method described by Botsoglou et al. [41] and the results expressed in mg MDA (malondialdehyde)/kg. The total volatile basic nitrogen (TVB-N) was measured by direct distillation of the homogenized samples according to the EU Commission Decision 95/149/EC [42] and the values expressed in mg/100 g of product. All analyses were performed in triplicate.

### 2.5. Microbiological Analyses

The microbiological analysis was performed as described by Messina et al. [19].

### 2.6. Sensorial Analysis

A sensory analysis on cold-smoked meagre fillets was conducted by a group of six trained judges according to an adapted version of the scheme proposed by Bilgin et al. [43] on hot-smoked meagre.

The parameters evaluated were appearance, odor, flavor, texture and color. The six judges rated the overall acceptability of the samples using a 10-point descriptive scale. A score of 9 to 10 indicates a perfect product, 7 to 8 good, 5 to 6 medium and 3 to 4 the limit of acceptability. The product is considered unacceptable when scoring less than 3 [43].

### 2.7. Statistical Analysis

The results were expressed as the mean ± standard deviation. Homogeneity of the variance was analyzed by Levene’s test. The data were analyzed by one-way analysis of variance (ANOVA), and Student–Newman–Keuls and Games–Howell post hoc tests were performed to make multiple comparisons between the experimental groups. Differences were statistically significant when *p* < 0.05. All data were analyzed by the SPSS for Windows^®^ application (version 15.0, SPSS, Chicago, IL, USA).

## 3. Results and Discussion

### 3.1. Physical–Chemical Parameters

Smoking is a process that alters the physical and chemical properties of the raw material, such as color, texture, pH, aw, water holding capacity (WHC), etc. It is important to control the physical and chemical parameters in the processed product, as they affect the shelf life and sensory characteristics [44].

#### 3.1.1. Color

Color is an important attribute of fish quality and freshness and also indicative of the chemical components and sensory attributes of food [45].

The results of the instrumental color analysis, in terms of L*, a* and b*; C* and h coordinates, obtained from *A. regius* fillets, are showed in Table 1.

As for the L* parameter (Table 1), it was observed that the cold-smoking process significantly affected the lightness, as both S and SA fillets showed a significant decrease (*p* < 0.05) of this parameter immediately after smoking. This is due to the effect of smoking, which induces a loss of water, which could have led to an increase in the carotenoid concentration and a decrease in hue and lightness [22,46,47,48].

During the shelf life, the L* parameter remained constant and increased significantly (*p* < 0.5) in the last days of storage (Table 1). This significant increase was also observed in other species such as sea bass [33].

Significant differences (*p* < 0.05) were observed between the two treatments (S and SA) starting from the 14th day of storage; the S samples showed significantly higher L* values than the SA samples, probably due to a higher moisture content in the sample. In fact, the higher percentage of water contributes to the creation of refractive indices in the food matrix, which leads to a greater luminosity [49,50].

The parameter a* decreased significantly (*p* < 0.05) during the shelf life in both treatments (S and SA). This decreased redness, mainly caused by the smoking process, has also been reported in salmon [51]; in fact, a general tendency has been observed in cold-smoked salmon to be darker and less red [51].

Regarding parameter b*, it decreased significantly after smoking (*p* < 0.05), then increased during storage (Table 1).

This yellowness of the muscle may be due to the resulting darkening of the bloodline due to hemeprotein oxidation, associated with a corresponding reduction in a* [22,52].

As shown in the present study, a general trend in cold-smoked meagre fillets was observed, i.e., they were darker and less red but more yellowish than unprocessed fillets. This yellowness was more evident in fillets treated with the natural antioxidant added during salting. Probably the coloration of the antioxidant affected the coloration of the final product.

#### 3.1.2. Texture Profile Analysis

The synergistic action of salt incorporation, the preservative effect of smoke compounds and dehydration during the smoking process can preserve fish. This leads to an increase in the tissue parameters, such as hardness [19,22,48,53,54,55,56,57].

**Figure 2:**
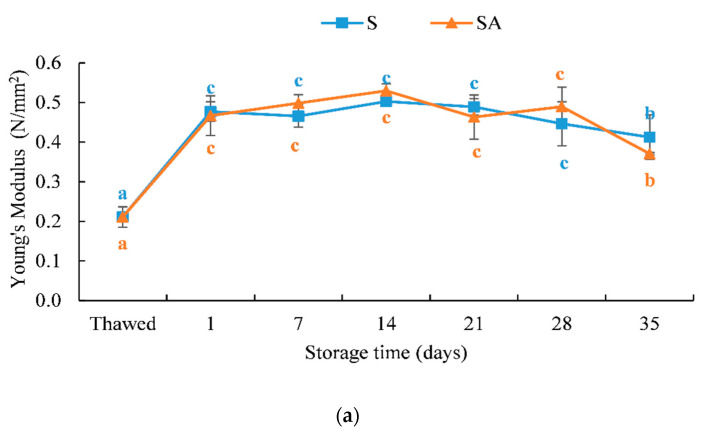

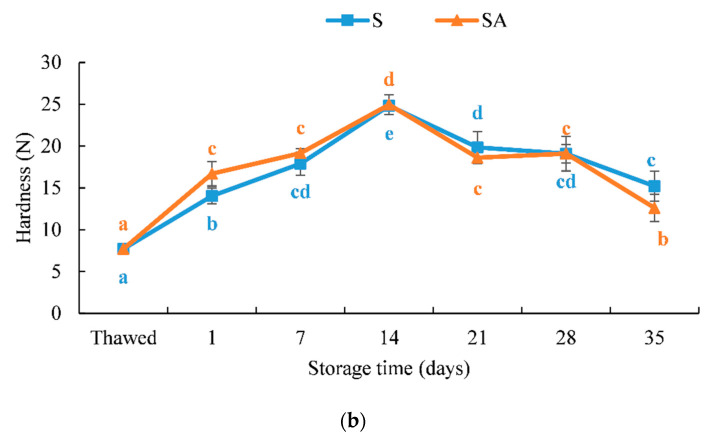
Results of the texture of cold-smoked meagre samples during storage at 4 ± 1 °C. (**a**) Young’s modulus and (**b**) hardness. Means with different letters (a, b, c and d) indicate significant differences (*p* < 0.05) during the shelf life for each single treatment.

The results of the texture analysis (Young’s modulus and hardness) are shown in Figure 2.

Young’s modulus (or modulus of deformability) values (Figure 2a) increased significantly (*p* < 0.05) after cold smoking and remained constant until T 28; a significant decrease (*p* < 0.05) was observed at T 35. No significant differences were observed between the two treatments.

The hardness value (Figure 2b) increased significantly until the 14th day of storage (Table 1), but no significant differences were observed between the treatments.

The increase in hardness was due to the synergistic effect of salting and drying causing meat hardening [53].

From the 21st day of storage, the hardness decreased significantly (*p* < 0.05) (Figure 2b); this decrease was probably due to the initiation of the degradation processes, mainly related to autolytic phenomena and protein denaturation in muscle tissue, resulting in a progressive reduction in tissue hardness [19,58,59].

#### 3.1.3. Water Holding Capacity (WHC)

The WHC is considered one of the most important parameters for preserving the fish quality and can influence the appearance and texture of fresh and processed fish products and, thus, the sensory quality of food [51,60,61].

**Figure 3:**
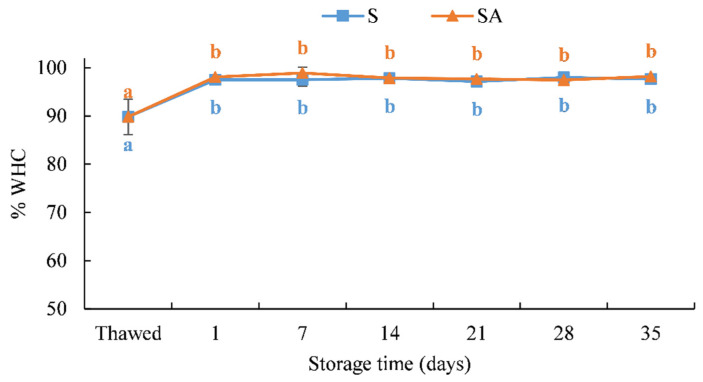
Results of the WHC% of cold-smoked meagre samples during storage at 4 ± 1 °C. (Means with different letters (a, b) indicate significant differences (*p* < 0.05) during the shelf life for each single treatment.

Changes in the WHC detected in fresh and smoked meagre during their shelf life are shown in Figure 3.

The smoking process resulted in a significant (*p* < 0.05) increase in the WHC in both treatments (Figure 3). This finding is in accordance with the results obtained in other cold-smoked species [19,22,54]. As observed by some authors [19,22,54], the increase in the WHC in smoked products is the result of the increase in the salt content. Indeed, as it is shown in Table 2 after cold smoking, an increase in the percentage of ash in the muscle was measured as a consequence of the salt uptake in the fish muscles.

The WHC remained stable throughout the storage time, with no significant differences between the two treatments.

#### 3.1.4. Muscular pH

**Figure 4:**
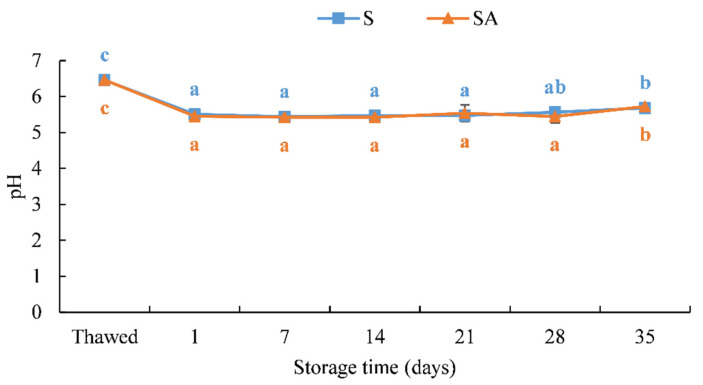
Results of the pH of cold-smoked meagre samples during storage at 4 ± 1 °C. (Means with different letters (a, b and c) indicate significant differences (*p* < 0.05) during the shelf life for each single treatment.

Changes in the pH values of cold-smoked meagre fillets during its shelf life are shown in Figure 4.

The smoking process significantly reduced the pH (*p* < 0.05). In fact, a significant difference was observed between the untreated and smoked samples (*p* < 0.05) (Figure 4).

The reduction in pH after smoking is related to the absorption of smoke acids; moisture loss and the reactions of phenols, polyphenols and carbonyl compounds with protein and amine groups [54,62]. In addition, the presence of salt, which causes an increase in the ionic strength of the solution within the cells, also contributes to the decrease in pH [54,63].

During the shelf life, it was observed that the pH remained constant in both treatments (Figure 4) and increased significantly on the last day of storage (day 35) with values of 5.69 ± 0.14 (S) and 5.73 ± 0.19 (SA).

The increase in pH can be attributed to the production of basic volatile components such as ammonia, trimethylamine and total volatile nitrogen by fish spoilage bacteria [22,64,65].

#### 3.1.5. Water Activity Determination

**Figure 5:**
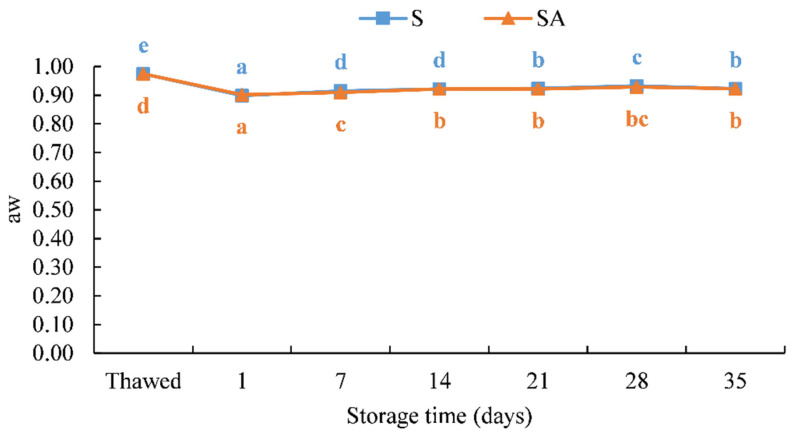
Results of the water activity (aw) of cold-smoked meagre samples during storage at 4 ± 1 °C. (Means with different letters (a, b, c, d and e) indicate significant differences (*p* < 0.05) during the shelf life for each single treatment.

The smoking process led to a decrease in the aw values detected in meagre fillets (Figure 5), which is also related to moisture reduction and an increase in the ash and mineral contents, as also observed in other species [22,54].

Normally, the aw factor in fish is close to 1, a value that was also observed in thawed meagre fillets (Figure 5). Fish processing can determine the reduction in aw values, reaching even values of 0.8 and 0.7 after heavy salting and drying [20].

The decrease in aw, due to osmotic pressure, results in the lower activity of bacteria and enzymes [66].

### 3.2. Proximate Composition and Biochemical Parameters Related to the Shelf-Life

#### 3.2.1. Proximate Composition

The proximate composition of untreated and smoked meagre fillets is shown in Table 2.

The lipid content showed that *A. regius* is a low-fat fish species [67] (Table 2).

In fact, farmed meagre has a much lower muscle fat than more commonly farmed Mediterranean fish species, including sea bream and European sea bass [68].

The relative contents of ash, water and lipids changed following smoking due to water loss. Salting and smoking determined a reduction in moisture and increase in the ash and mineral contents, as observed in previous studies on other fish species, such as European sea bass and dolphinfish [19,22,54].

**Figure 6:**
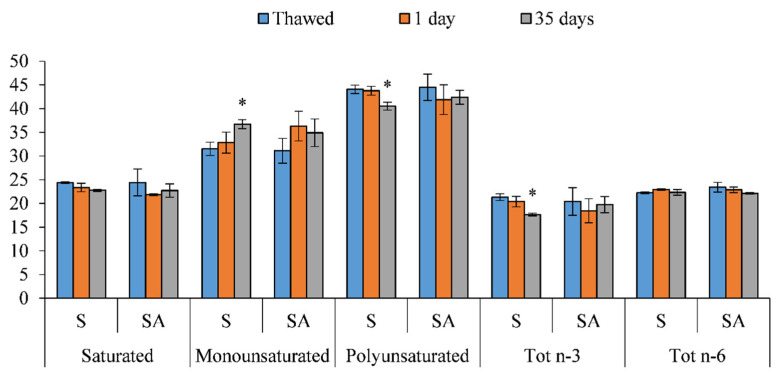
Fatty acid classes of thawed and cold-smoked meagre samples during storage at 4 ± 1 °C (* *p* < 0.05).

Figure 6 shows the fatty acid profile of smoked meagre fillets.

The role played by the natural antioxidant in preserving the oxidation of the fatty acids was evident. In fact, S fillets (Figure 6) showed a significant reduction in polyunsaturated fatty acids (*p* < 0.05) at the end of smoking (35 days) due to a decrease in polyunsaturated fatty acids of the n-3 series (Tot n-3) (*p* < 0.05) and a simultaneous increase in monounsaturated fatty acids (Figure 6). In SA smoked sea bass fillets, Tot n-3 remained unchanged during the shelf life, as did the other classes of fatty acids.

#### 3.2.2. Biochemical Parameters Related to the Shelf-Life

The antioxidant treatment of fillets of different species has been shown to prevent lipid oxidation [19].

Malondialdehyde (MDA) is a secondary lipid oxidation product. As observed in other fish species, such as dolphinfish (*C. hippurus*), a lower lipid content, combined with a high polyunsaturated fatty acid content, could lead to a greater susceptibility to peroxidation than other fish species, such as sardines (*S. pilchardus*), which have a higher lipid content but a higher saturated fatty acid content, which are less susceptible to oxidation [27].

**Figure 7:**
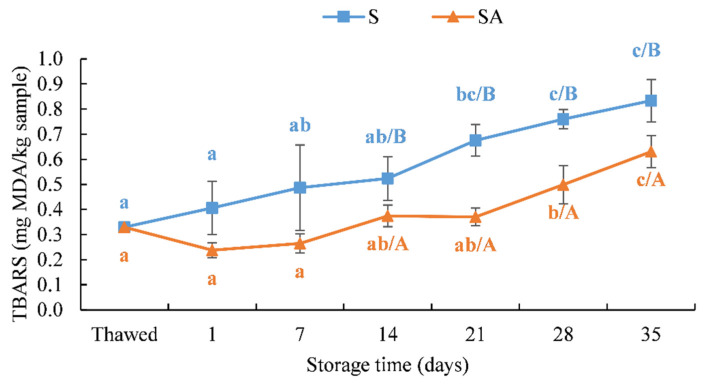
Results of the TBARS (determined as mg MDA/kg samples) of cold-smoked meagre samples during storage at 4 ± 1 °C. (Means with different letters (a, b and c) indicate significant differences (*p* < 0.05) during the shelf life for each single treatment. Means with different letters (A, B) indicate significant differences (*p* < 0.05) between the two treatments at the same storage time.

As shown in Figure 7, the SA treatment determined a marked reduction in the MDA content (mg MDA/kg) in smoked meagre fillets compared to the S treatment, which produced a higher MDA content after 7 days of refrigerated storage. The levels of the MDA results were statistically different between the treatments (*p* < 0.05) (Figure 7) from 7 days until the end of the trial.

The low MDA content was detected in sous vide meagre (*A. regius*) fillets treated with natural antioxidants during cold storage, and the MDA values did not exceed the limit value during the storage period [69]. In that study, low values of MDA (0.52 ± 0.13-mg MDA/kg) were detected in the raw fillets, and lower values of MDA were found during cold storage in fillets treated with antioxidants compared to the control, highlighting that the application of natural antioxidants in the meagre sous vide process could have had positive effects on the lipid peroxidation process [69].

Moreover, the results obtained in the present study in terms of the low MDA content detected in smoked fillets during storage agreed with those obtained in another farmed species, i.e., European sea bass (*D. labrax*), whose fillets were treated by cold smoking, highlighting the preservative effect of the process on this biochemical parameter related to the fish shelf life [22].

The results obtained in the present study also agreed with what was obtained in previous studies, in which the antioxidant treatment combined with smoking was effective in preventing lipid peroxidation [19,27]. Indeed, the combined treatments markedly reduced the MDA content in dolphinfish fillets up to 35 days of storage compared to cold smoking used alone as a preservative treatment [19]. Moreover, as in the case of sardines (*S. pilchardus*), a fatty fish species, the lipid oxidation results showed that the combined application of oregano extract and smoking significantly reduced the oxidation (*p* < 0.05) in sardines in comparison with the batch that was only smoked [27].

It has to be highlighted that, in both cases, cold-smoked fillets did not exceed the 4 to 5-mg MDA/kg value considered acceptable for smoked fish in the literature [65]. Moreover, vacuum packaging might be effective in preserving processed products from lipid oxidation, from a technology hurdle point of view, providing more evidence on the effective use of these methods of preservation [70,71].

**Figure 8:**
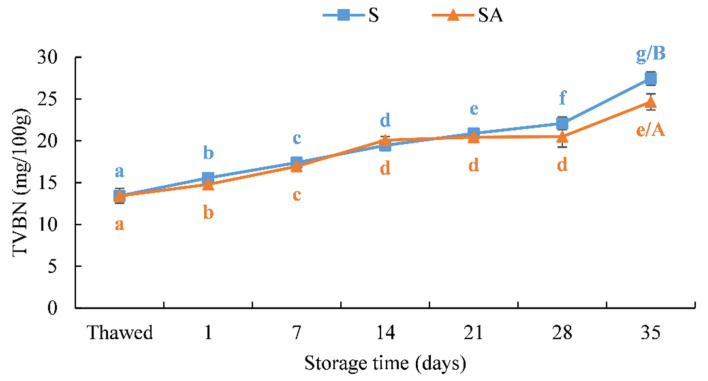
TVBN contents in cold-smoked meagre fillets during storage at 4 ± 1 °C. (Means with different letters (a, b, c, d, e, f and g) indicate significant differences (*p* < 0.05) during the shelf life for each single treatment; means with different letters (A, B) indicate significant differences (*p* < 0.05) between the two treatments at the same storage time.

The total volatile basic nitrogen (TVB-N) values detected in smoked meagre fillets during storage are shown in Figure 8. European legislation has established an upper limit for TVB-N, ranging from 25 to 35-mg TVB-N/100 g [42]. Nevertheless, considering that a TVB-N threshold for processed fish products has not been established so far, a limit equal to 35-mg TVB-N/100 g has been considered for smoked fish products [19,25]. In the present study, this threshold was not exceeded during the preservation period and up to 35 days of storage in both batches (SA and S) (Figure 7). Nevertheless, it has to be highlighted that, at T35, the level of TVB-N content in the SA batch was lower than that observed in the S batch, with their values significantly different (*p* < 0.05).

The TVB-N may be affected by a reduction in both the spoilage bacteria and the activity of endogenous enzymes [72]. The findings obtained in the present study agreed with what was obtained in previous studies, which highlighted the effectiveness of the salting/smoking processes on maintaining the TVB-N content below the threshold for spoilage during refrigerated storage under vacuum or modified atmosphere packaging [19,22,73].

### 3.3. Microbiological Analyses

**Figure 9:**
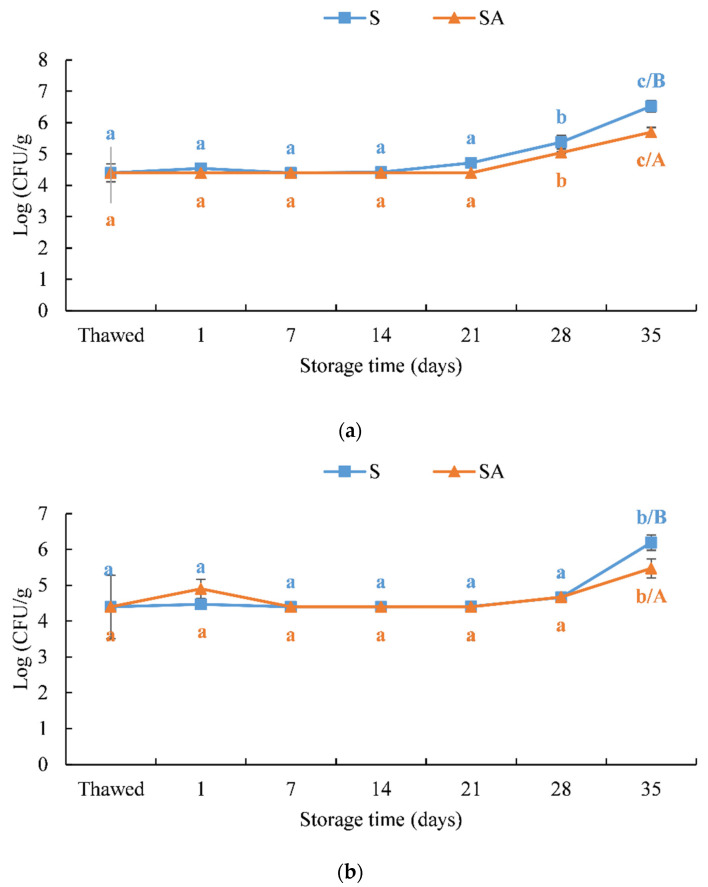
Microbiological evaluation during the shelf life of cold smoked meagre fillets stored at 4 ± 1 °C. (**a**) Mesophilic (37 °C) and (**b**) psychrophilic (6 °C). Means with different letters (a, b and c) indicate significant differences (*p* < 0.05) during the shelf life for each single treatment. Means with different letters (A and B) indicate significant differences (*p* < 0.05) between the two treatments at the same storage time.

Microbiological analyses highlighted the bacteriostatic effect of cold smoking combined with the natural antioxidant. This effect is due to the synergistic action of the salt uptake during the brining phase and the polyphenols deposition during the smoking phase [19,25,74]. In thawed meagre fillets, the bacterial load was 4.40 ± 0.29 log (CFU/g) for mesophilic bacteria and 4.40 ± 0.89 log (CFU/g) for psychrophilic bacteria (Figure 9). During storage, the bacterial load remained constant in both treatments up to 21 and 28 days of storage for mesophilic bacteria and psychrophilic bacteria, respectively. At the end of storage, a significant increase in both mesophilic and psychrophilic bacteria was observed (Figure 9) in the two treatments, although the values did not exceed 10^7^ CFU/g, according to the current standard [75,76,77,78,79].

### 3.4. Sensorial Analysis

The results from the sensory evaluation were strictly related to those of the physical–chemical and biochemical parameters considered to determine the fish quality and freshness during the shelf-life trials and give important findings about consumer perception and acceptability [19,25,80]. In the present study, both the S and SA batches did not show any differences for any of the sensory attributes evaluated, showing that the method of processing and the use of antioxidants from the halophyte *H. strobilaceum* effectively preserved the quality and shelf life of meagre [19]. The sensory quality evaluation showed that the smoked fillets of meagre were of good quality until 35 days of refrigerated storage under vacuum packaging (Table 3).

As far as odor and taste were concerned, the sensory evaluation performed confirmed what was obtained from the biochemical analyses in terms of the TVB-N and MDA contents. In particular, even if significant differences were observed between the batches in terms of the MDA contents, the minimum value of 1.44-mg MDA/kg detectable by the panelists [81] was not exceeded in both batches up to the end of storage (Figure 6).

## 4. Conclusions

*A. regius* is today one of the new frontiers of aquaculture. Our work demonstrated the validity of processing this species through cold smoking with the use of natural antioxidants, which could therefore represent a means of obtaining an innovative yet safe product.

In fact, the combined treatment with cold smoking and antioxidant resulted in an overall improvement in the quality characteristics and shelf life of meagre fillets; the antioxidant treatment prevented the lipid peroxidation and degradation of the nitrogen components.

From a sensory and technological point of view, the presence of antioxidants of natural origin did not lead to detectable changes in the final product.

## Figures and Tables

**Table 1:** Results of the color of cold-smoked meagre samples during storage at 4 ± 1 °C.

	Storage Time (Days)	S	SA
L* (D65)	Thawed	45.48 ± 1.43 ^c^	44.65 ± 1.41 ^d^
	0	40.86 ± 1.26 ^a^	39.87 ± 1.13 ^b^
	1	40.52 ± 0.57 ^a^	39.46 ± 0.18 ^b^
	7	39.17 ± 0.67 ^a^	38.87 ± 0.10 ^ab^
	14	40.96 ± 1.03 ^a/B^	37.96 ± 0.41 ^a/A^
	21	40.14 ± 0.18 ^a/B^	38.36 ± 0.07 ^a/A^
	28	39.90 ± 0.76 ^a^	39.84 ± 0.59 ^b^
	35	42.72 ± 0.33 ^b/B^	41.38 ± 0.39 ^c/A^
a* (D65)	Thawed	−2.12 ± 0.39 ^d^	−2.31 ± 0.43 ^c^
	0	−2.35 ± 0.28 ^cd^	−2.72 ± 0.31 ^bc^
	1	−2.57 ± 0.07 ^c/B^	−2.85 ± 0.10 ^bc/A^
	7	−3.47 ± 0.36 ^b^	−3.55 ± 0.12 ^ab^
	14	−3.29 ± 0.03 ^b^	−2.63 ± 0.79 ^b^
	21	−4.31 ± 0.02 ^a/A^	−3.50 ± 0.10 ^ab/B^
	28	−3.61 ± 0.06 ^b/A^	−3.09 ± 0.09 ^b/B^
	35	−3.31 ± 0.39 ^b/B^	−4.25 ± 0.10 ^a/A^
b* (D65)	Thawed	−3.56 ± 0.83 ^b^	−2.61 ± 0.75 ^a^
	0	−6.67 ± 0.59 ^a/A^	−3.90 ± 0.68 ^a/B^
	1	−5.62 ± 0.63 ^b/A^	−2.80 ± 0.58 ^a/B^
	7	−3.41 ± 0.96 ^c/A^	−1.05 ± 0.01 ^b/B^
	14	−2.20 ± 0.13 ^cd/A^	0.37 ± 0.37 ^c/B^
	21	−0.75 ± 0.64 ^d^	0.43 ± 0.22 ^c^
	28	−0.14 ± 0.54 ^d^	1.41 ± 0.59 ^c^
	35	−1.68 ± 0.79 ^cd/A^	1.51 ± 0.08 ^c/B^
C* (D65)	Thawed	4.21 ± 0.84 ^a^	3.53 ± 0.82 ^ab^
	0	7.09 ± 0.62 ^b/B^	4.78 ± 0.69 ^b/A^
	1	6.25 ± 0.53 ^b/B^	4.04 ± 0.47 ^ab/A^
	7	4.91 ± 0.92 ^a^	3.82 ± 0.05 ^ab^
	14	4.16 ± 0.23 ^a^	2.81 ± 0.67 ^a^
	21	4.53 ± 0.16 ^a/B^	3.68 ± 0.16 ^ab/A^
	28	3.80 ± 0.01 ^a^	3.50 ± 0.31 ^ab^
	35	3.93 ± 0.01 ^a/A^	4.60 ± 0.14 ^b/B^
h (D65)	Thawed	238.17 ± 4.55 ^d^	226.24 ± 5.82 ^cd^
	0	250.57 ± 1.60 ^e/B^	234.51 ± 3.37 ^d/A^
	1	244.05 ± 2.92 ^d/B^	222.83 ± 5.22 ^c/A^
	7	222.51 ± 5.36 ^c/B^	195.21 ± 0.43 ^b/A^
	14	211.64 ± 0.05 ^bc/B^	167.86 ± 10.09 ^a/A^
	21	188.24 ± 7.93 ^a^	172.47 ± 3.32 ^a^
	28	180.02 ± 8.95 ^ab^	156.66 ± 8.67 ^a^
	35	206.35 ± 13.97 ^bc/B^	161.40 ± 0.16 ^a/A^

The means with different letters (a, b and c) in the same column are significantly different (*p* < 0.05). The means with different letters (A, B and C) in the row are significantly different (*p* < 0.05).

**Table 2:** The proximate composition (g/100 g) of thawed and cold-smoked meagre samples during storage at 4 ± 1 °C.

	Storage Time (Days)	S	SA
Total Lipids	Thawed	0.85 ± 0.06 ^a^	0.88 ± 0.12 ^a^
	1	1.72 ± 0.26 ^b^	2.15 ± 0.23 ^b^
	35	2.25 ± 0.26 ^b^	2.51 ± 0.39 ^b^
Moisture	Thawed	79.57 ± 1.74 ^b^	80.57 ± 1.79 ^b^
	1	74.96 ± 1.54 ^a^	74.19 ± 1.05 ^a^
	35	73.03 ± 1.17 ^a^	73.08 ± 1.71 ^a^
Protein	Thawed	18.06 ± 1.24	17.04 ± 1.18
	1	18.18 ± 0.10	18.02 ± 0.10
	35	18.84 ± 0.53	18.84 ± 0.81
Ash	Thawed	1.26 ± 0.07 ^a^	1.23 ± 0.07 ^a^
	1	5.13 ± 0.88 ^b^	5.22 ± 0.68 ^b^
	35	5.10 ± 0.15 ^b^	4.67 ± 0.88 ^b^

The means with different letters (a, b) in the same column are significantly different (*p* < 0.05).

**Table 3:** Results of the sensory analysis of cold-smoked meagre samples during storage at 4 ± 1 °C.

	Days	S	SA
Appearance	1	9.00 ± 0.82 ^bcd^	9.00 ± 0.00 ^bcd^
	7	10.00 ± 0.00 ^d^	9.25 ± 0.50 ^d^
	14	8.25 ± 0.50 ^abc^	8.75 ± 0.50 ^bcd^
	21	8.00 ± 1.15 ^abc^	7.75 ± 0.50 ^ab^
	28	8.00 ± 0.89 ^abc^	8.17 ± 0.41 ^abc^
	35	7.17 ± 0.75 ^a^	7.00 ± 0.89 ^a^
Color	1	9.25 ± 0.96 ^cd^	8.75 ± 0.50 ^bc^
	7	10.00 ± 0.00 ^d^	9.25 ± 0.50 ^c^
	14	8.50 ± 0.58 ^bc^	8.75 ± 0.50 ^bc^
	21	8.50 ± 0.58 ^bc^	8.25 ± 0.96 ^b^
	28	8.17 ± 0.98 ^bc^	8.50 ± 0.55 ^b^
	35	7.33 ± 0.52 ^ab^	7.00 ± 0.89 ^a^
Odor	1	9.50 ± 0.58 ^d^	8.00 ± 0.00 ^b^
	7	9.50 ± 0.58 ^d^	9.25 ± 0.50 ^b^
	14	8.50 ± 0.58 ^bcd^	9.00 ± 0.82 ^b^
	21	8.50 ± 0.58 ^bcd^	8.00 ± 0.82 ^b^
	28	7.83 ± 0.75 ^bc^	8.17 ± 0.41 ^b^
	35	7.17 ± 0.75 ^ab^	6.67 ± 1.03 ^a^
Texture	1	9.75 ± 0.50 ^c^	7.50 ± 0.58 ^a^
	7	9.75 ± 0.50 ^c^	9.00 ± 0.00 ^bc^
	14	9.00 ± 0.00 ^bc^	9.75 ± 0.50 ^c^
	21	8.50 ± 0.58 ^b^	8.25 ± 0.96 ^b^
	28	9.00 ± 0.00 ^bc^	9.00 ± 0.00 ^bc^
	35	7.33 ± 0.52 ^a^	7.00 ± 0.89 ^a^
Taste	1	9.50 ± 0.58 ^b^	8.25 ± 0.50 ^bc^
	7	9.50 ± 0.58 ^b^	9.25 ± 0.50 ^c^
	14	8.50 ± 0.58 ^ab^	9.00 ± 0.82 ^c^
	21	8.25 ± 0.96 ^ab^	7.25 ± 0.96 ^ab^
	28	7.83 ± 0.75 ^a^	8.17 ± 0.41 ^bc^
	35	7.17 ± 0.75 ^a^	6.67 ± 1.03 ^a^

The means with different letters (a, b, c and d) in the same column are significantly different (*p* < 0.05).

## Data Availability

Not applicable.
